# Optimized Metal Chalcogenides for Boosting Water Splitting

**DOI:** 10.1002/advs.201903070

**Published:** 2020-04-06

**Authors:** Jie Yin, Jing Jin, Honghong Lin, Zhouyang Yin, Jianyi Li, Min Lu, Linchuan Guo, Pinxian Xi, Yu Tang, Chun‐Hua Yan

**Affiliations:** ^1^ State Key Laboratory of Applied Organic Chemistry Key Laboratory of Nonferrous Metal Chemistry and Resources Utilization of Gansu Province College of Chemistry and Chemical Engineering Lanzhou University Lanzhou 730000 China; ^2^ Department of Chemistry Brown University Providence RI 02912 USA

**Keywords:** electronic structure, enhanced catalysis, metal chalcogenides, synthetic methods, water splitting

## Abstract

Electrocatalytic water splitting (2H_2_O → 2H_2_ + O_2_) is a very promising avenue to effectively and environmentally friendly produce highly pure hydrogen (H_2_) and oxygen (O_2_) at a large scale. Different materials have been developed to enhance the efficiency for water splitting. Among them, chalcogenides with unique atomic arrangement and high electronic transport show interesting catalytic properties in various electrochemical reactions, such as the hydrogen evolution reaction, oxygen evolution reaction, and overall water splitting, while the control of their morphology and structure is of vital importance to their catalytic performance. Herein, the general synthetic methods are summarized to prepare metal chalcogenides and different strategies are designed to improve their catalytic performance for water splitting. The remaining challenges in the research and development of metal chalcogenides and possible directions for future research are also summarized.

## Graphical abstract

Herein, the general synthetic methods and different strategies for preparing metal chalcogenides are summarized to improve their catalytic performance in the hydrogen evolution reaction and oxygen evolution reaction. The challenges of built metal chalcogenides catalysts are summarized to enhance and develop future promising applications.

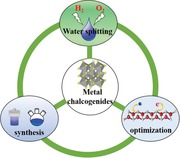

## 1 Introduction

Nonrenewable fossil fuels, like coal, oil, and natural gas, afford more than 80% of the global energy consumption. However, the emission during the combustion of fossil fuels cause serious environmental issues like global warming and air pollutions. With renewable electricity from solar, wind, and hydropower, this process is emission‐less and energy efficient, and therefore is an important part in the development of low‐carbon economy.^1^, ^2^, ^3^, ^4^, ^5^ Electrocatalytic water splitting (2H_2_O → 2H_2_ + O_2_), consisting of hydrogen evolution and oxygen evolution reaction (HER/OER), can convert electricity to chemical energy in H_2_ and O_2_ for further energy applications. The practical application of overall water splitting, however, is still limited due to the lack of effective and stable catalysts to reduce reaction energy barrier and enhance Faraday efficient for both reactions.^6^, ^7^, ^8^, ^9^, ^10^ Different materials have been studied for overall water splitting catalysis, like metal chalcogenides, metal carbides, oxides, etc. The transition metal carbides, especially graphene, have a better electroconductivity, ductility, and high surface area which display excellent performance in water splitting.^11^, ^12^, ^13^ Oxides as another abundant species on the earth also show better water splitting performance, but their stability in hard media is not very good.^14^, ^15^, ^16^ The layered double hydroxides (LDH) with unique structure, abundant interstratified electrons and channels for intermediate adsorption and desorption display wonderful water splitting performance.^17^, ^18^, ^19^, ^20^ Additionally, the metal–organic frameworks (MOFs) and their based nanocrystals as newly nanomaterials have got much attention in various fields, but their structure limited the active sites exposure for the complete coordinative metal sites.^21^, ^22^, ^23^, ^24^, ^25^ Therefore, it is important to enable the cost‐effective, large‐scale production of these catalysts, and further improve the performance and efficiency of overall water splitting.

**Scheme 1:**
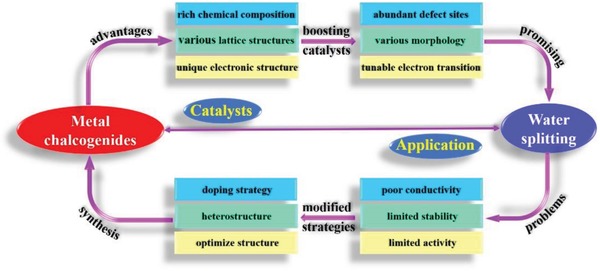
The metal chalcogenides applied in water splitting.

Transition metal chalcogenides have many different compositions with various lattice structure, while those materials also have unique electronic structures.^26^ Based on those superior properties, the transition metal chalcogenides show promising application in many energy applications,^27^ such as electrochemical catalysis, photocatalysis, metal–air batteries, and other energy conversion reactions. Especially for their abundant defects sties,^26^, ^27^, ^28^ tunable electronic structure,^29^, ^30^, ^31^, ^32^ and various morphology,^33^, ^34^, ^35^, ^36^, ^37^ the transition metal chalcogenides exhibit boosting performance for water splitting. However, they still have some disadvantages, such as poor conductivity, activity, and stability, in water splitting limited their large‐scale industrial application.^38^, ^39^, ^40^, ^41^, ^42^ How to synthesize the active and stable transition metal chalcogenides is still a big challenge for wide application. In this Review, the several promising strategies are designed to prepare the active and stable transition metal chalcogenides (**Scheme** **1**).

**Scheme 2:**
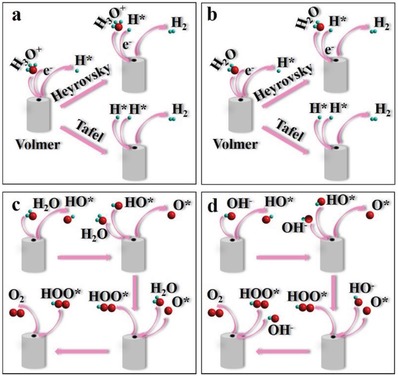
The mechanism of HER in a) acid and b) alkaline media (b). The mechanism of OER in c) acid and d) alkaline media.

HER and OER as the two important half‐reactions consist the overall water splitting to produce highly pure H_2_ and O_2_. In generally, the overall water splitting needs a minimum voltage of 1.23 V to obtain the gas products, but the actual potential for water splitting is much higher than the theoretical data due to the energy loose in the electrochemical system and poor activity of the electrocatalysts.^8^, ^9^ Therefore, many efforts have been devoted to enhance the efficiency of the whole system and prepare active and stable electrocatalysts.^11^, ^15^, ^20^, ^25^ The most active catalysts for HER are platinum (Pt) based materials, while the iridium (Ir), ruthenium (Ru), and their oxides show superior OER performance, but the high price of those noble metal and limited stability make them application at the commercial scale is still challenging.^43^, ^44^, ^45^, ^46^, ^47^ Recently, various transition metal‐based catalysts exhibit excellent performance for HER and OER, especially for those metal chalcogenides show higher activity than noble metal‐based catalysts for OER.^48^, ^49^, ^50^ More importantly, in this review the basic mechanism for HER and OER in both acid and alkaline media are summarized (**Scheme** **2**). The HER progress has similar steps under acid (Scheme 2a) or alkaline media (Scheme 2b). The first step for HER is Volmer reaction with H_3_O^+^ to form H* in acid media and H_2_O to form H* in alkaline media, respectively. The second step is rate determining step (RDS) depends on the activity of those catalysts, such as Pt‐based catalysts with Tafel reaction adsorbed H* and H* to form H_2_, while the transition metal catalysts usually show Heyrovský reaction with H_3_O^+^ (acid) or H_2_O (alkaline) and H* to form H_2_. The reason for different reaction pathway can be attributed to free energy of hydrogen adsorption (Δ*G*_H*_) for Pt is close to zero, while the Δ*G*_H*_ for transition metal composition is much higher. Therefore, combined with density functional theory (DFT) methods, the preparation of transition metal‐based catalysts with low Δ*G*_H*_ is still challenge in this field. OER is a more complex reaction with four‐electrode transition and many intermediates (HO*, O*, and HOO*) (Scheme 2c,d). In generally, there are four pathways for formation O_2_ (H_2_O (acid)/OH^−^ (base) → HO* → O* → HOO* → O_2_, each step with one electron transition) under any condition.^48^, ^49^ With the different activity, catalysts show different free energy for each pathway, while the step that requires the most energy become RDS. More importantly, with the development of the technology, the surface oxidation on various catalysts and lattice oxygen mechanism becomes the focus of research.

**Figure 1:**
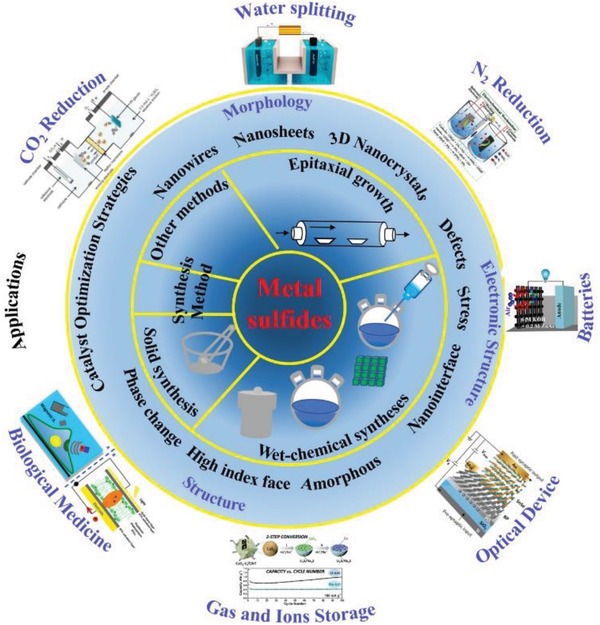
Illustration of typical synthesis and application of metal chalcogenides.

In particularly, metal chalcogenides, especially metal sulfides, have been intensively researched for the application in hydrodesulfurization (HDS) reaction.^51^, ^52^, ^53^, ^54^, ^55^ With the similar mechanism of HDS and HER with H_2_ dissociation to produce H_2_S in HDS and H* adsorbed on catalyst to create hydrogen for HER, it is believed that metal chalcogenides may be active for HER. Recently, the researches for HER were focused on enhancing the conductivity and active sites for catalysts. Molybdenite, molybdenum disulfide (MoS_2_), and MoS_2_ based nanomaterials as the most famous catalysts for HER are widely used in industry today.^54^, ^55^ It has a variety of morphologies and shows different catalytic properties. For example, the ultrathin MoS_2_ with atomic layer thickness, especially for 1T‐MoS_2_, demonstrates comparable HER catalytic activity to Pt, in which the excellent activity can be attributed to abundant edges defects.^46^, ^47^, ^48^, ^49^, ^50^, ^51^, ^52^, ^53^, ^54^, ^55^, ^56^, ^57^, ^58^, ^59^ The surface sites,^60^, ^61^, ^62^, ^63^ defects,^64^, ^65^ disulfide linkages, and triangular MoS_2_ units^66^ can be shown amazing catalytic performance. Ni_3_S_2_, main components of mineral heazlewoodite, is intrinsic metallic for the abundant Ni—Ni bonds in the structure.^67^, ^68^, ^69^, ^70^ With high conductivity and low cost, Ni_3_S_2_ has been used in kinds of electrochemical reactions,^67^, ^68^, ^69^, ^70^, ^71^, ^72^ but their activity is less competitive than noble‐metal catalysts.^73^, ^74^, ^75^, ^76^, ^77^ Generally, the pyrite‐type transition metal disulfide (FeS_2_) with abundant S_2_^2−^ ions show fast electrical transport and have employed as promising catalysts for electrocatalytic reactions,^78^, ^79^, ^80^, ^81^, ^82^ but the lack of basic active sites limited their intrinsic activity.^83^, ^84^, ^85^, ^86^, ^87^, ^88^ Besides, metal chalcogenides can also be made into various heterostructures and nanocomposites, which show enhanced catalysis in electrochemical reactions (**Figure** **1**).^96^, ^97^, ^98^, ^99^, ^100^, ^101^, ^102^, ^103^, ^104^, ^105^, ^106^, ^107^, ^108^, ^109^, ^110^, ^111^, ^112^, ^113^, ^114^, ^115^, ^116^

In this review, we provide a summary of metal chalcogenides for water splitting. First, we introduce current methods to synthesize different types of metal chalcogenides. Second, we discuss different strategies employed to tune the morphology, crystallinity, defects, interface, phase, crystal facets, stress, and heterostructures of metal chalcogenides, and their effects on water splitting catalysis. Last, we indicate the challenges in the research and development of metal chalcogenides as the water splitting catalysts, and our perspectives on the future directions to solve the problems.

## 2 Synthesis of Metal Chalcogenides

**Figure 2:**
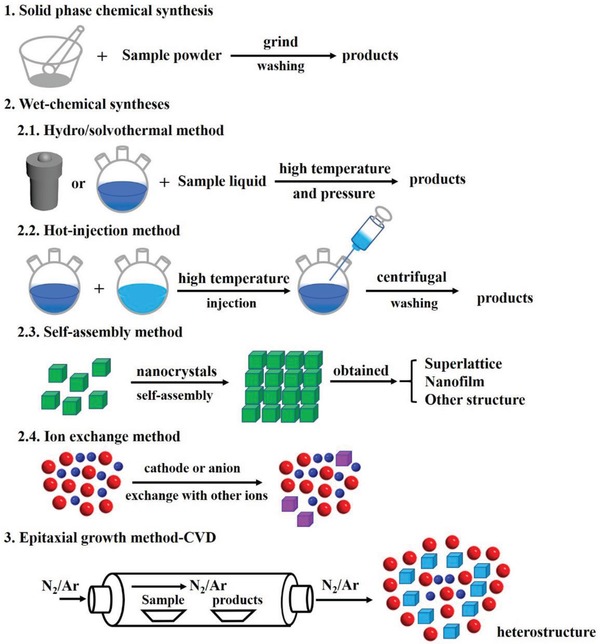
The principal methods for synthesizing metal chalcogenides.

The most special characteristics of metal chalcogenides, which benefits from their controlled morphology and abundant active sites, is the high‐activity, making them ideal choose for construction of other hybrid nanomaterials.^53^, ^117^, ^118^, ^119^, ^120^, ^121^, ^122^, ^123^, ^124^ In general, metal chalcogenides can be prepared through solid phase chemical synthesis, wet‐chemical synthesis, and chemical vapor deposition (CVD) based epitaxial growth method. As shown in **Figure** **2** and **Table** **1**, different methods can be applied in engineering multifunctional metal chalcogenides and metal chalcogenides‐based hybrid nanostructures to tune their physical, and chemical properties to adjust for various applications.^125^, ^126^, ^127^, ^128^, ^129^, ^130^, ^131^, ^132^, ^133^, ^134^, ^135^ As a class of traditional nanomaterials, discover new features of metal chalcogenides and fabrication of hybridizing metal chalcogenides with other materials have been subjects of intensive studies in recent years. Till now, metal chalcogenides have been used in many fields, such as catalysts,^115^, ^116^, ^117^, ^118^, ^119^, ^120^, ^121^, ^122^, ^123^ battery,^53^, ^109^, ^110^, ^111^, ^116^, ^117^, ^118^, ^119^, ^120^, ^121^, ^122^, ^123^, ^124^, ^125^, ^126^, ^127^, ^128^ optical application,^136^, ^137^, ^138^, ^139^ biomedicine,^112^, ^124^, ^140^, ^141^, ^142^ gas and ions storage,^103^, ^143^, ^144^, ^145^, ^146^, ^147^, ^148^, ^149^, ^150^, ^151^, ^152^, ^153^, ^154^, ^155^, ^156^, ^157^ etc. Metal chalcogenides‐based hybrid nanostructures are also interesting, like metal chalcogenides coupling with noble metals,^55^, ^158^, ^159^, ^160^, ^161^, ^162^, ^163^ oxides,^33^, ^93^, ^109^, ^117^, ^124^, ^155^, ^164^, ^165^, ^166^, ^167^, ^168^, ^169^, ^170^, ^171^, ^172^, ^173^, ^174^, ^175^, ^176^, ^177^, ^178^, ^179^, ^180^, ^181^, ^182^, ^183^, ^184^, ^185^, ^186^, ^187^, ^188^, ^189^, ^190^, ^191^, ^192^, ^193^ carbon materials,^194^, ^195^, ^196^, ^197^, ^198^, ^199^, ^200^, ^201^, ^202^, ^203^ etc.^204^, ^205^, ^206^, ^207^, ^208^ And the design of functionalized metal chalcogenides and metal chalcogenides‐based hybrid nanostructures is very important to the catalytic performance in HER and OER.

**Table 1:** The summary of advantages and disadvantages of various synthetic methods of metal chalcogenides

Methods	Advantages	Disadvantages
Solid phase Chemical synthesis	Low cost Simple equipment and process Rapid reaction rate	Low purity Uneven morphology
Hydro/solvothermal synthesis	High selectivity and efficiency Good dispersion Controlled morphology	Unclear reaction mechanism Use of surfactants Time‐consuming
Hot‐injection method	Uniform size and morphology High crystallinity Controlled morphology	Small organic molecules residuals removal Low yield
Self‐assembly of nanocrystals	Wide application Ordered multistage nanostructure Rapid reaction rate	Delicate control of the reaction process High‐quality nanocrystals required
Ion exchange method	High selectivity and efficiency Wide range of application Rapid reaction rate	Relatively low Crystallinity Difficult to process
Chemical vapor deposition (CVD)	High efficiency for 2D nanocrystals Multiheterostructure engineering	Specific equipment required High temperature Limited application

### 2.1 Solid Phase Chemical Synthesis

Solid phase chemical synthesis, especially under low temperature, has attracted increasing research interest for its high selectivity and efficiency.^113^, ^209^ It is generally agreed that the solid phase chemical synthesis include four stages: diffusion, reaction, nucleation, and growth. And the rate‐determining step varies from case to case. Compared with the gas phase or liquid phase reaction, the mechanism of the solid phase chemical synthesis is more complicated and special. And different from liquid and gas phase reactions, the concentration of solid matter change is not used to promote the kinetic is of the solid phase chemical synthesis. In the solid phase reaction, the reaction energy mainly comes from lattice vibrations, defects motion, and the migration of ions and electrons. The products obtained by the solid phase chemical synthesis show unique crystal structure and morphology, energy states, and internal defects.^209^ Therefore, carefully researches of the solid phase chemical synthesis should be done for its complexity and particularity. In general, the most widely used research method for solid phase chemical synthesis should be thermal decomposition. There are two forms for the solid phase chemical synthesis, which can be divided into nucleation and kinetics progress. In the crystal structure, the active center can be formed in the location with absence of symmetry and internal defects for the different coordination environment. The nucleation reaction first occurred on those active centers, forming the initial reaction nucleus. Then, the kinetics reaction happens, which is depended on the nuclear formation rate, growth rate, and expansion rate. In generally, the activation energy of nucleus is greater than that of growth activation, so once the nucleus is formed, it can grow and expand rapidly. The application of powder reaction is also very extensive, but it is affected by many factors, such as particle size, size distribution, morphology, homogeneity of material mixture, contact area, function relationship between the phase number of the reaction and time, and vapor pressure and evaporation rate of the powder. Additionally, it is also necessary to consider the size and distribution of granularity, degree of loading, and contact area, if the reaction happened at a lower temperature.

### 2.2 Wet‐Chemical Syntheses

#### 2.2.1 Hydro/Solvothermal Synthesis

The hydro/solvothermal synthesis, as one of the most famous wet‐chemical synthesis strategy, is widely used in preparation materials.^208^ The boiling point of the solvents (water or organic solvents) is usually lower than the reaction temperature, as the temperature increases, high pressure is built up in the closed system, promotes the reaction and improves the crystallinity. In this method, both surfactants and reactants can be sulfur sources. With his method, metal chalcogenides with different properties (detailed properties, e.g., metal chalcogenides with different shape, defects, and phases) can be obtained.

**Figure 3:**
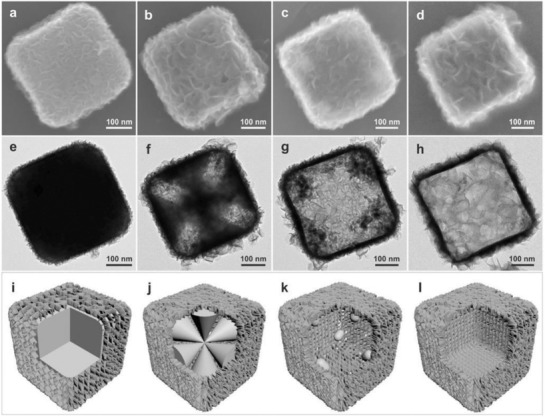
a−d) FESEM images and e−h) TEM images of the as‐synthesized products obtained at different time intervals: 1 h a,e), 6 h b,f), 12 h c,g), and 20 h d,h). i−l) Schematic illustration of the formation process of Ni−Co−MoS_2_ nanoboxes. Reproduced with permission.^129^ Copyright 2016, John Wiley‐VCH.

In the hydro/solvothermal approach, the interaction between host species and surfactants is critical for the formation of metal chalcogenides. By changing solvents, surfactants, and other additives like small ions (K^+^, Al^3+^, SO_4_^2−^, etc.), metal chalcogenides with different size, morphology and structure can be obtained. For example, Xie and co‐workers used the solvothermal method and prepared freestanding 1.4 nm thick CoSe_2_ nanosheets with abundant Co vacancies, which shows an overpotential of 0.32 V at 10 mA cm^−2^ in of OER 0.1 m KOH solution.^84^ Lin and co‐workers prepared the hierarchical NiCo_2_O_4_ hollow microcuboids with bifunction catalytic properties for both HER and OER.^210^ Additionally, the NiCo_2_O_4_ electrodes also show activity toward overall water splitting with a potential of 1.65 and 1.74 V for 10 and 20 mA cm^−2^. Solvothermal method as one of the simplest and most effective methods has been applied to synthesis metal chalcogenides with different morphology. In generally, the reaction uses metal oxides or metal hydroxides as precursor in thiourea or thioacetamide solution under sealed condition reacted for several hours. Using this strategy, various metal chalcogenides, such as NiCo_2_S_4_, FeS_2_, CoS, MnS, and other metal chalcogenides with controllable morphology was developed. MOF was also used as precursors to prepare metal chalcogenides. Lou and co‐workers synthesized a nickel and cobalt incorporated molybdenum disulfide (MoS_2_) hollow nanoboxes with enhanced electrochemical activity for HER (**Figure** **3**).^129^ They show very prefect morphology and structure for those hollow nanoboxes (Figure 3a–h) and the possible reason for formation of the hollow structure is also given (Figure 3i–l).

Various nanostructure of MoS_2_ has been developed and applied in many fields especially for HER, which exhibits an excellent HER performance demonstrated by both computationally and experimentally. Additionally, many researches indicate that edges in MoS_2_ nanostructure are the initial HER active sites.^49^, ^50^, ^51^, ^52^, ^53^, ^54^, ^55^, ^56^, ^57^, ^58^, ^59^, ^60^, ^61^, ^62^, ^63^, ^64^, ^65^, ^66^ Therefore, many efforts are developed to prepare MoS_2_ with more exposed edge sites and improved HER activity. For example, with controllable disorder and oxygen incorporation engineering, Xie and co‐workers fabricated an optimized MoS_2_‐based catalyst with onset‐potential of 120 mV and excellent stability for HER.^174^ Cui and co‐workers reported the heteroatoms (Fe, Co, Ni, Cu)‐doped MoS_2_ nanofilms shows increased exchange current density.^26^

However, the growth period is difficult to observe in hydro/solvothermal progress due to the closed system, making it challenging to study monitor the process and modify experimentally. The hydro/solvothermal synthesis is quite sensitive to the experimental conditions, which means that the researchers should focus on concentration of precursor, solvent, surfactants, polymers, and temperature making a precise control in every batch and laboratory. For example, most of the 2D nanosheets prepared by the hydro/solvothermal synthesis method were a few‐layers thick, while single‐layer nanosheets can be prepared by what method. To gain more control of this method, research can be done to 1) understand the complicated mechanism; 2) design larger reaction devices and optimize the reaction process; 3) improve the efficiency of the postprocess; and 4) improve the uniformity of the same batch products.

#### 2.2.2 Hot‐Injection Method

**Figure 4:**
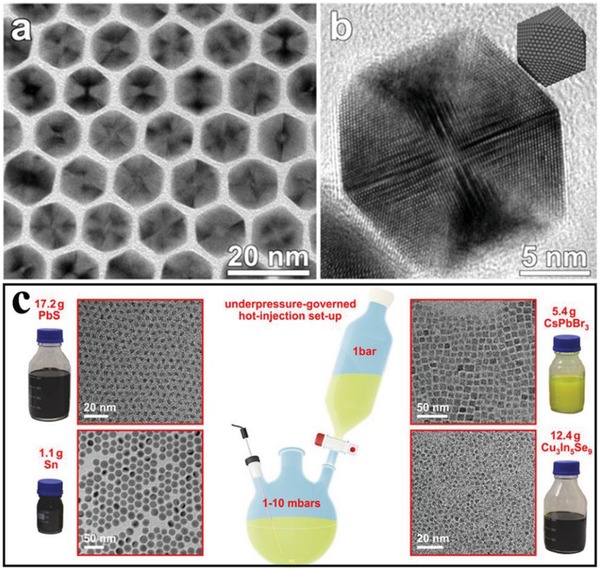
a) TEM and b) HRTEM micrograph of Pt icosahedra with an edge length of 8.8 nm. Reproduced with permission.^211^ Copyright 2013, American Chemical Society. c) Schematic illustration of hot‐injection methods and TRM for the obtained products. Reproduced with permission.^212^ Copyright 2016, American Chemical Society.

The hot‐injection route is a common method to prepare monodisperse colloidal nanocrystals with controllable size, shape, and composition.^201^, ^202^, ^203^, ^204^, ^205^, ^206^, ^207^, ^208^ Generally, this method is starting with rapid injection of the reactants into the hot reaction solution containing key surfactants (oleylamine, oleic acid, etc.). Yang and co‐workers prepared uniform 8.8 nm Pt icosahedral nanocrystals via the hot‐injection method (**Figure** **4**a,b), which shows an area specific activity of 0.83 mA cm^−2^ for oxygen reduction reaction (ORR).^211^ Then, the authors studied the influence of nucleation rate, growth kinetic, and atmosphere on the finally shape of the nanocrystal, which indicating that the icosahedral crystals created under oxygen gas atmosphere. Wood and co‐workers developed pressure‐assisted hot‐injection method for the fabrication of metallic (Sn) and semiconductor (PbS, CsPbBr_3_, and Cu3In_5_Se_9_) nanocrystals (Figure 4c).^212^ The technical details of this under‐pressure assisted hot‐injection method for the large‐batch syntheses are also described. On one hand, high‐quality nanoparticles can be obtained with the hot‐injection method. On the other hand, this method also shows some limits. First, long‐chain surfactants are usually used in the reactions and difficult to remove afterward, which have a bad effect in catalysis, batteries, and other energy storage reactions. Second, high temperature is usually required to convert metal precursor to metal sulfide, limiting the solvent choice to be high boiling point only. Third, the quality of nanocrystals is better controlled with lab‐scale synthesis and the scale‐up may require some extra efforts.

#### 2.2.3 Self‐Assembly of Nanocrystals

**Figure 5:**
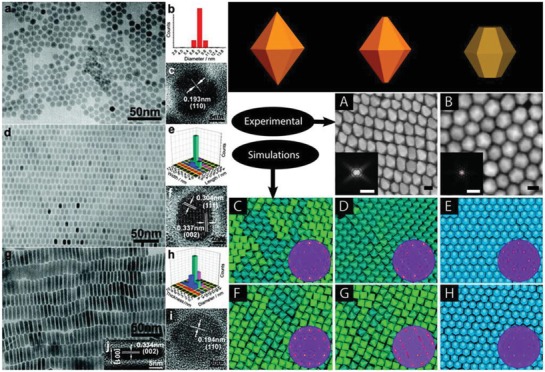
a–c) TEM image, size distribution, and HRTEM image of circular Cu_2_S nanocrystals. d–f) TEM image, size distribution, and HRTEM image of elongated Cu_2_S nanocrystals. g–i) TEM image, size distribution, and HRTEM image of Cu_2_S hexagonal nanoplates. Reproduced with permission.^215^ Copyright 2008, American Chemical Society. Schematics of the particle geometries at top panel in right. SEM images of self‐assembled superlattices of hexagonal bipyramid‐shaped ZnS NCs A) and hexagonal bifrustum‐shaped ZnS NCs B). Snapshots of 2D self‐assembly of hexagonal bipyramids C,D,F,G) and hexagonal bifrustums E,H) adhered to an air−toluene interface. Reproduced with permission.^216^ Copyright 2014, American Chemical Society.

Self‐assembly of nanocrystals is that presynthesized nanocrystals spontaneously arrange themselves into an ordered structure based on van der Waals interactions, electrostatic interactions, and hydrogen bonds between nanocrystals.^213^, ^214^, ^215^, ^216^ It is an efficient way to create nanoarchitectures with combined or integrated catalytic functionality. Many researchers have been made intensive study in self‐assembly strategy about nanocrystal fabrication and application. In this year, Maspoch and co‐workers used ZIF‐8 based dodecahedral particles fabricated the 3D rhombohedral lattice thought the self‐assemble.^213^ With optimized photonic bandgap and sensitive response for adsorption, those superstructures show the promising applications in sensing. Zheng and co‐workers reported 1D Pd superlattice nanowires by face‐to‐face assembly of surfactant‐free Pd nanosheets.^214^ In the experiment, they used cations with higher charge density to decreased electrostatic repulsion between Pd nanosheets with negatively charged, which is of great importance in assembling Pd nanosheets based superlattice nanowires. More and more researches indicating that the dipole moment, small positive charge, and directional hydrophobic attraction plays an important role in the self‐assembly reaction. Additionally, the self‐assembly strategy as an emerging method has been widely used in the synthesis of metal chalcogenides. Li and co‐workers reported the highly ordered Cu_2_S multilayer superlattices via self‐assembled the highly uniform Cu_2_S obtained through a simple water–oil interface confined reaction (**Figure** **5**a–j).^215^ This work provides a simple bottom‐up approach to fabricate various self‐assembled metal chalcogenides superlattices structure thought controlling the size and shape of the presynthesized block nanocrystals for integrate nanocrystals, as well as their properties for the potential applications in catalysts and energy conversion devices. Similarly, Donega and co‐workers present a self‐assembly ZnS 2D superlattices with hexagonal bipyramid and hexagonal bifrustum morphology (Figure 5A–H).^216^ The analysis shows that interfacial free‐energies and packing density driven the self‐assembly, while the morphology structure has an important influence to assembly structure, which highlighting the important of precise morphology control in self‐assembly field.

#### 2.2.4 Ion Exchange Method

The ion exchange method is used to tune the composition of the metal chalcogenides by replacing the existing ions with other ions, which is a common way in the fabrication of series hybrid structure nanomaterials, such as metal oxides, LDHs, metal phosphides, and metal chalcogenides.^188^, ^189^, ^190^, ^191^, ^192^, ^193^ The ion exchange process usually generates defects, nanointerface, or strain in the nanocrystals, which may introduce some changes to the electrocatalytic properties.

##### Cation Exchange Method

**Figure 6:**
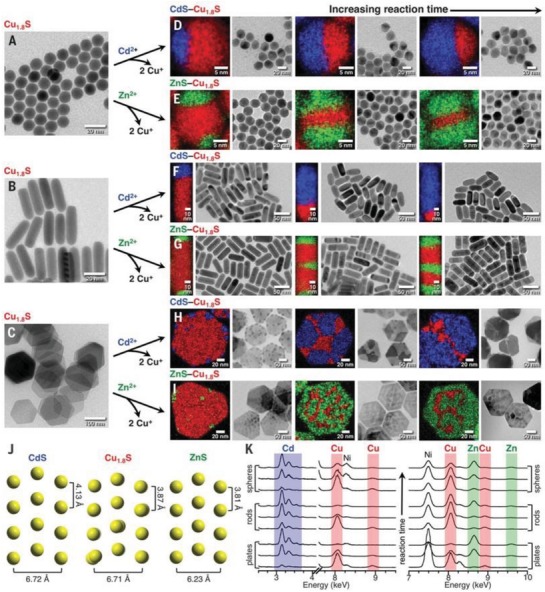
A–I) Formation of various Cu_1.8_S based nanostructures with different morphology through cathode exchange method. Cu, Cd, and Zn are shown in red, blue, and green in TEM images and STEM‐EDS elements maps. J) Crystal structure projections of wurtzite CdS, roxbyite Cu_1.8_S, and wurtzite ZnS, which highlight the crystallographic relationships between the adjacent phases and intraparticle frameworks. K) EDS spectra of selected regions for all samples. The Ni signal is from the Ni TEM grid. Reproduced with permission.^193^ Copyright 2018, AAAS.

The cation exchange is a simple and efficient process usually applied to compound various nanocrystals. The cation exchange can induce novel structure in as‐synthesized nanocrystals, including hybrid structure, heterostructure and core−shell structure.^188^, ^189^, ^190^, ^191^, ^192^, ^193^ However, the exact pathways of those cations are still remaining unknown in the formation progress of the new nanocrystals. The new phase created after cation exchange reactions often shows the lowest‐energy phase and exhibits better performance for electrocatalysis and energy conversion reaction. Alivisatos's group reported a series of CdS nanocrystals preparation based on the cation exchange method.^217^, ^218^, ^219^ In 2007, they reported the heterogeneous superlattices structure consisting of CdS nanorods and Ag_2_S dots through partial cation exchange of Cd^2+^ by Ag^+^.^217^ The successfully fabricated CdS−Ag_2_S nanocrystal, indicating the lattice‐mismatch strains may also happened in colloidal nanocrystal growth. The CdS−Ag_2_S superlattices structure with high stability displays a promising application in nanometer‐scale optoelectronic devices. Additionally, the transition cation exchange reactions also have attracting significant attention in recent years.^160^ Robinson and co‐workers reported the dual‐interface heterostructure based on Cu*_x_*S nanocrystals through cation exchange.^191^ In this system, the solid–solid phase transformation started from copper sulfide phase to lower‐energy phase, and finally came back to copper sulfide phase for the epitaxial alignment with the formed material. More importantly, in this year many perfect works about precious synthesis chemistry have been reported. Schaak and co‐workers prepared a series metal sulfide based on asymmetric, patchy, porous, and sculpted control (**Figure** **6**).^193^ Using cation exchange method, 0D, 1D, and 2D of copper sulfide nanocrystals can be obtained (Figure 6A–C). Those heterostructure nanocrystals with precisely defined materials and interfaces will be important for many applications. Although the more and more nanocrystals have been synthesized by cation exchange method, the control of cation transport pathway and quantity still needs further study. Additionally, Schaak's group developed colloidal nanocrystal cation exchange reactions for 3d transition metal systems to study the transformation of Cu_2−_ *_x_*S into CoS and MnS, which indicating that both anion and cation sub‐lattice features can be retained during nanocrystal cation exchange reactions.^220^ Those works provide useful guidelines for predictably targeting desired structural features in nanocrystal line metal chalcogenides and for selectively accessing one of multiple phases in polymorphic solid‐state systems.

##### Anion Exchange Method

**Figure 7:**
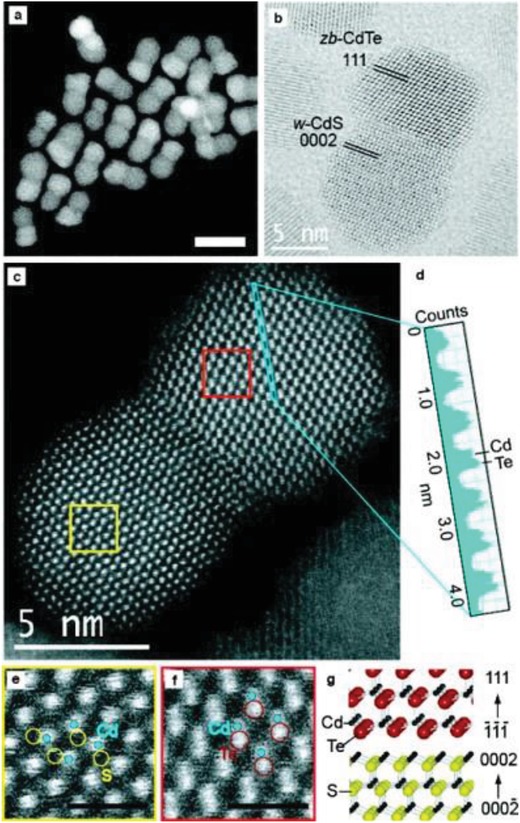
a–c) HAADF‐STEM, BF‐STEM images, and atomic‐resolution HAADF‐STEM image of CdS/CdTe heterodimer. d) Z‐contrast profile of anion/cation pair columns in the blue rectangular region in (c). e,f) Magnified images of the yellow and red rectangular regions for e) CdS and f) CdTe phases in (c), respectively. Scale bars = 1 nm. g) Illustration of the CdS/CdTe heterointerface. Reproduced with permission.^228^ Copyright 2011, American Chemical Society.

Recently, the researches indicating that nanocrystals with mesoporous structures or hollow interiors should possess excellent performance in many fields for the high specific surface areas and active sites in those nanomaterials. Among various synthesis methods, ion exchange method has been proven very effective way for fabricating the hollow structural. More importantly, Kirkendall effect gives an unambiguous interpretation about the anion transfer path and formation of hollow structure.^127^, ^129^ Therefore, the ion‐exchange process may bring a new way to create complex hollow structures for application in various fields. Various compounds like perovskite, metal chalcogenides and phosphides, and other novel compounds have been developed by anion exchange method. Lou's group reported series work about anion exchange method for fabrication various nanocrystals with hollow structure, such as CoS_2_ nanobubble hollow prisms,^221^ onion‐like NiCo_2_S_4_ particle,^222^ CoS hollow structures,^223^ nitrogen‐doped carbon@NiCo_2_O_4_ double shelled nanoboxes,^224^ ultrathin Ni‐Fe LDH nanosheets composed hierarchical hollow nanoprisms,^225^ and so on. In the synthesis, A precursor is prepared and then followed by a sulfidation in the corresponding reactions. The Kirkendall effect clearly demonstrates the formation of hollow structure with faster outward diffusion of A anion than inward diffusion of S. These hollow structures, due to high surface are and optimized adsorption, show enhanced catalytic activity in HER and OER. Heterostructures can be made from the anion exchange method as well.^226^, ^227^ Teranishi and co‐workers synthesized anisotropic CdS/CdTe heterodimers for the different crystal structures of CdS and CdTe (**Figure** **7**) via the exchange between S and Te.^228^ The heterointerfaces in CdS/CdTe heterodimers affect what property of metal chalcogenides, leading to what interesting applications.

Similarly, Zhang's group preparedCo_3_FeS_1.5_(OH)_6_ hydroxychalcogenides by immersing Co‐based hydroxide into solution with high‐concentration of S^2−^ anions.^229^ They show an electrocatalytic performance with low OER potential (η_j = 10_ = 1.588 V) and ORR half‐wave potential (0.721 V), indicating a high potential gap (0.867 V) for reversible oxygen electrocatalysis. Further, they also developed the application of those catalysts and batteries and fabricated the self‐driven water splitting device.^17^ This method can be a promising method for wide synthesize the other hydroxysulfides with excellent electrocatalytic performance for OER, HER, and ORR.

#### 2.2.5 Other Wet‐Chemical Synthesis Methods

Metal chalcogenides, as an important inorganic component, also can be prepared by other wet‐chemical synthesis methods. Li and co‐workers reported the sulfur and nitrogen‐codoped carbon loaded single atoms of Fe (Fe‐ISA/SNC) catalysts by a novel pyrrole−thiophene copolymer pyrolysis strategy with superior catalytic activity for ORR with low half‐wave potential (0.896 V) and long‐term stability (15 000 cycles),^230^ which reveal that the enriched charge of N/S surrounding the Fe reactive center and improved ORR efficiency. Lewis and co‐workers prepared MoSe_3_ film through an operando method of synthesis with interesting HER performance.^231^ During the electrocatalysis, the as‐prepared smooth MoSe_3_ film was converted to MoSe_2_ with pores ≈200 nm in diameter with low overpotential (η_j = 10_ < 50 mV) and remarkable stability (T > 48 h). Additionally, electrochemical deposition as another efficient method have widely used for synthesis various nanocrystals. Electrodeposition has its unique advantages, such as convenient, fast, and low cost, but it still has several disadvantages namely poor reproducibility and controllability. Sun and co‐workers synthesized cobalt‐sulfide film via electrochemical deposition on conductive substrates as a highly efficient electrode for hydrogen generation in neutral media with low onset potential (43 mV) and Tafel slope (93 mV dec^−1^). It also shows a good performance in photoelectrochemical hydrogen generation due to what property of this metal chalcogenides. With the development of technology and mechanism, the newly methods should be further designed to prepare various metal chalcogenides.

In summary, the liquid phase chemical synthesis of the metal chalcogenides is easy to control, and high‐quality nanocrystals can be obtained. However, there are still some disadvantages for the liquid phase chemical, which can be classified as 1) the mechanism of this system is complex and inconvenient research; 2) the function of various liquid in the system also is ambiguous; 3) the precise control about fabrication specific surface or interface is difficult; 4) ions transition, liquid maxing, and the reaction mechanism on different interface is not clear; 5) the system is easy to be polluted and the separation of liquid and products is complicated.

### 2.3 CVD Based Epitaxial Growth Method

**Figure 8:**
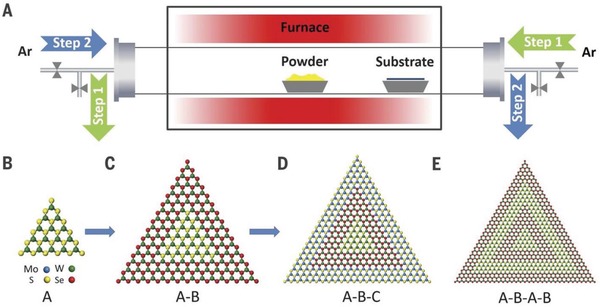
A) Schematic illustration of a modified CVD system for the robust epitaxial growth of lateral heterostructures. The solid powders were directly used as the source material. The obtained various 2D nanocrystals monolayer seed (A,B), A‐B heterostructure (C), A‐B‐C multiheterostructure (D), and A‐B‐A‐B superlattice (E). Reproduced with permission.^237^ Copyright 2017, AAAS.

The CVD method is considered as a reliable and robust method to prepare various ultrathin 2D metal chalcogenides. In generally, the substrate and cycled gas/vapor precursors are put in the furnace chamber, in which the corresponding reactions take place on the surface of substrate and obtain the 2D materials. Zhang's group reported many works about CVD method prepared metal chalcogenides and studied their application in various fields, such as electrocatalysis, sodium storage, lithium‐ion batteries, and so on.^133^, ^232^, ^233^, ^234^, ^235^, ^236^ The ultrathin 2D metal chalcogenides prepared through CVD process usually possess high crystallinity with less defects and high purity. The size and thickness can be controlled by different parameters. With the developing of the CVD method, the epitaxial growth method was designed to prepare ultrathin 2D heterostructures, especially for those 2D metal chalcogenides. Recently, various 2D nanocrystals with superior morphology and structure, for example ultrathin nanosheets, heterostructures, superlattices, and multiheterostructures, have been developed via epitaxial growth. However, the obtained nanomaterials usually must growth on the substrate such as SiO_2_/Si, fluoride‐doped tin oxide, tin‐doped indium oxide, and so on, and their large‐scale application in industry remains a challenge. Duan and co‐workers reported a newly epitaxial growth method through modified step‐by‐step CVD process (**Figure** **8**).^237^ In the new CVD system, the uniquely designed angle‐style at both ends of quartz tube can change the direction of argon gas flow. Additionally, both gas inlet and outlet are equipped on the two sides of the quartz tube. The reaction includes two steps. The step 1 was temperature ramping and stabilization stage for the formation of precursor steam, and step 2 started at the growth temperature with flow argon transport the precursor steam onto substrate for epitaxial growth newly unique 2D nanocrystals. Combine monolayer seed (B) with precursor steam (A) can obtain a series superior nanostructure were synthesized (Figure 8C–E). Generally, in traditional epitaxial growth mechanism, the lattice symmetry and lattice constant of 2D nanocrystals and substrate usually display a better match. However, in the van der Waals epitaxial growth progress the heterostructure of 2D nanocrystals can fabricated without lattice symmetry even with different crystalline and large lattice disorder. Zhang's group used similar method and fabricated the metallic VSe_2_ nanosheets with high electrical conductivity of 106 S m^−1^.^238^ The obtained VSe_2_ nanosheets with different thickness from several nanometers to several tenths of nanometers possess various charge‐density wave phase transitions. Although the various 2D nanomaterials have been prepared through epitaxial growth method, the wide application of those materials in different fields still a challenge. Additionally, the products obtained by CVD method shows high purity, clear surface, and interface structure and easy control to synthesis heterostructure products with different species. But the method still has some deficiencies need improvement, such as low production rate, difficult in preparation smaller size products, and maintain product consistency in widely production.

## 3 Various Strategies Applicated in Metal Chalcogenides for Water Splitting

Various catalysts demonstrate important roles in water splitting. Recently, metal chalcogenides offer an alternative to noble metal catalysts owing to their low cost, easy of fabrication, and intrinsic electronic structure for water splitting (Figure 2). The most striking characteristics of metal chalcogenides, which benefits from their rich component and electronic structure, is adjustable active sites, making them ideal objects for construction of highly efficient electrocatalysts. A number of metal chalcogenides materials have been demonstrated to generate functional hybrid nanoarchitectures though different optimizing strategy, such as morphology (1D, 2D, 3D, and other morphology), structural control (porous structure, phase statue regulation, amorphous structure), electronic structure optimization (defects, vacancy, nanointerface, stress regulation), heterostructure with other nanomaterials (oxides, hydroxides, carbon materials, noble metals), and so on (**Table** **2**).^49^, ^50^, ^51^, ^52^, ^53^, ^54^, ^55^, ^56^, ^57^, ^58^, ^59^, ^60^, ^61^, ^62^, ^63^, ^64^, ^65^, ^66^, ^67^, ^68^, ^69^, ^70^, ^71^, ^72^, ^73^, ^74^, ^75^, ^76^, ^77^, ^78^, ^79^, ^80^, ^81^, ^82^, ^83^, ^84^, ^85^, ^86^, ^87^, ^88^, ^89^, ^90^, ^91^, ^92^, ^93^, ^94^, ^95^ It is noteworthy that despite that they possess an excellent electrocatalytic performance for water splitting, the morphology, structure, valence state and content change of the elements, and electronic properties (conductivity, bandgap, density of states) of those metal chalcogenides should be carefully confirmed after electrocatalytic reaction of water splitting due to the inevitable oxidation will happen on the surface of the electrocatalysts during OER, and the reduction reaction will also happen under HER condition. Therefore, an in‐depth study should be made to confirm the electrocatalytic performance changes of those surface modified metal chalcogenides. In this section, various kinds of metal chalcogenides‐composites had been developed and used in water splitting, and the surface change of those catalysts were also confirmed during the reaction progress.

**Table 2:** The summary of advantage and disadvantage of various strategies for metal chalcogenides in the overall water splitting

Strategies		Advantage	Disadvantage
Morphology	1D structure	Anisotropy Easy preparation	Low surface area Limited activity
	2D structure	High atomic efficiency Abundant edge active sites Defects	Difficult synthesis Rapid reaction rate
	3D structure	High specific surface area Porous	Low stability Limited activity
Structure	Phase change	Changeless morphology and species Enhanced activity	Limited synthesis strategies
	High index face	High density of atomic steps, ledges, and kinks Excellent catalytic activity	Difficult in preparation
	Amorphous	Abundant defects High active	Low stability Low crystallization
Electronic structure	Defects	Excess active sites Favorable adsorption energy	Imprecise control Uncounted quantity
	Nanointerface	Strong coupling Lattice disorder	Lattice matching growth
	Stress	Low d‐band center of metal and Reduced adsorption of oxygen‐containing species	Difficult in characterization

### 3.1 Morphology

#### 3.1.1 1D Nanowires

**Figure 9:**
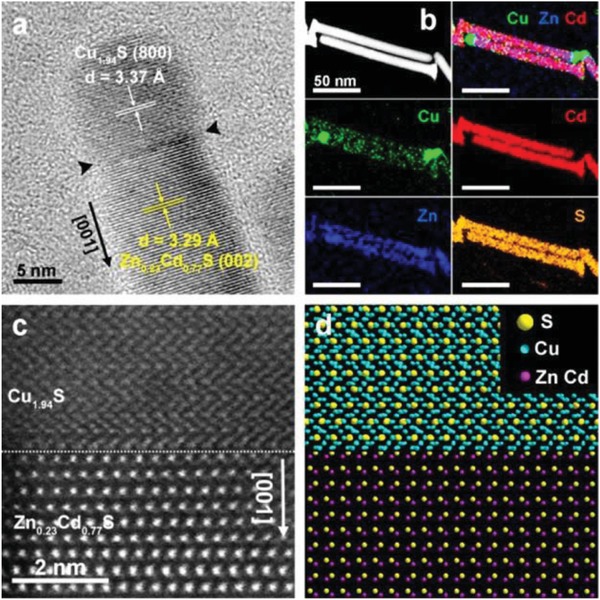
a) HRTEM and b) EDX‐STEM elemental mapping images, c) Atomic‐resolution aberration‐corrected HAADF‐STEM image, and d) the corresponding model of the Cu_1.94_S−Zn_0.23_Cd_0.77_S heterointerface. Reproduced with permission.^117^ Copyright 2016, American Chemical Society.

1D structures, such as nanowires (NWs) and nanotubes (NTs), exhibit unique advantages associated with their anisotropy, structure and surface properties compared with the corresponding nanoparticles, showing the good potentials for enhancing the activity and stability for water splitting.^6^ To be specific, the 1D nanomaterials possess fewer lattice boundaries, easier electron and mass transports, longer segments of smooth crystal planes grown along a certain crystal surface and controllable surface defect sites. Many researches indicating that Pt‐based nanowires show a superior electrocatalytic performance, but the prohibitive cost limited their widely application.^239^, ^240^, ^241^, ^242^ Therefore, newly abundant, nonprecious nanomaterials with better electrocatalytic activity should be developed to replace Pt‐based materials. Recently, with the development of the nanotechnology, the inherently anisotropic cheaper metal‐based 1D nanostructures with enhanced activity have been successfully synthesized. For instance, the 1D NWs arrays on carbon fiber paper, nickel foam (NF), copper foam, and other conductive subject not only possess the unique properties of NWs, but also have increased conductivity for the self‐supporting electrode without other conducting polymer, such as Nafion and carbon black, avoiding the carbon‐corrosion problem and displaying highly efficient for overall water splitting. Xia and co‐workers reported a metallic (Ni, Co)_0.85_Se nanotube arrays with unique structure and abnormally high concentration of defect sites, which exhibited better OER activity and stability than pure Co_0.85_Se.^243^ Li and co‐workers reported the Cu_1.94_S−Zn*_x_*Cd_1−_ *_x_*S heteronanorods (0 ≤ x ≤ 1) with highly active visible‐light photocatalyst performance (**Figure** **9**).^117^ With continuous composition adjustment, the Cu_1.94_S−Zn_0.23_Cd_0.77_S heteronanorods with the optimal bandgap achieve an efficient hydrogen production activity of 7735 µmol h^−1^ g^−1^. Nevertheless, despite the obvious advantages of 1D metal chalcogenides nanomaterials in activity and durability enhancement, the key challenge has been turned to the generation of active 1D nanostructures using reliable synthetic techniques.

#### 3.1.2 2D Nanosheets

**Figure 10:**
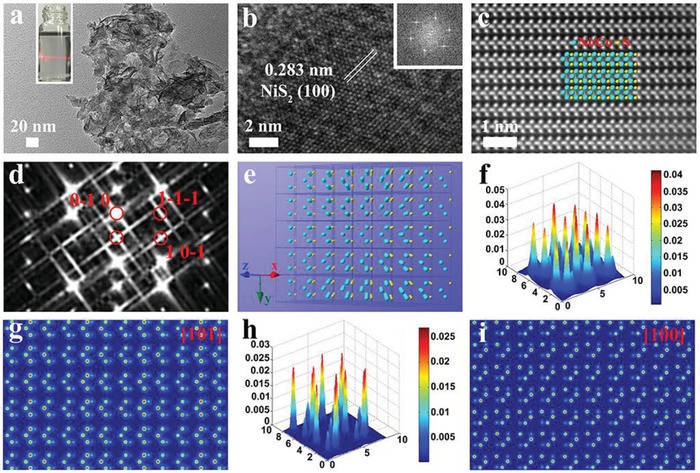
a) TEM, b) HRTEM, c) HAADF‐STEM images, and d) corresponding FFT image of HAADF‐STEM for Co‐NiS_2_ NSs. e) Crystal structure of Co‐NiS_2_ NSs depicting Ni/Co cations as blue spheres and S in orange; Surface intensity and atomic columns simulated by using QSTEM software along f,g) [101] and h,i) [100] zone axes. Reproduced with permission.^250^ Copyright 2019, Wiley‐VCH.

As an emerging new nanomaterial, the 2D nanosheets, especially ultrathin 2D nanostructure, have been getting an extensive attention in many fields, such as catalysis, optics, electricity. Generally, several unique advances of 2D nanomaterials for water splitting have been identified in past years. First, those 2D nanocrystals, especially for ultrathin 2D nanomaterials, are conducive to exposure the active sites with increased catalytic activity. Second, the strong in‐plane and out‐plane coupling between the atoms in the 2D nanostructure possess the unique electricity performance, which plays an important role in electrocatalysis. Third, 2D nanomaterial with ultrahigh specific surface area endows abundant absorption sites for reaction active species in the electrocatalysis.^133^, ^232^, ^233^, ^234^, ^235^, ^236^ Herein, we give a detailed discussion about the synthesis of those 2D nanocrystals. The obtained 2D nanocrystals possess abundant defects, such as vacancy, lattice disorder, distortion, especially prepared by liquid exfoliation method. Those newly species can be acting as excess active sites for various catalysis. Last, the surface functionalization may be an easy strategy to fabricate 2D nanocrystal‐based catalysts. The unique performance of 2D nanocrystals indicates that the careful characterization should also be developed. The transmission electron microscope (TEM) and atomic force microscope (AFM) are usually used to confirm the morphology of the 2D nanocrystals. The extended X‐ray absorption fine structure spectroscopy (EXAFS), X‐ray photoelectron spectroscopy (XPS), electron paramagnetic resonance, and positron annihilation spectroscopy (PAS) were used to prove the defects in those materials. Various materials with 2D nanostructure have been successfully fabricated, such as oxides, nitrides, chalcogenides, carbides, and so on. Zhang and Xie's groups have reported many amazing nanocrystals with 2D structure.^79^, ^84^, ^96^, ^97^, ^128^, ^131^, ^133^, ^232^, ^233^, ^234^, ^235^, ^236^, ^237^, ^238^, ^239^, ^240^, ^241^, ^242^, ^243^, ^244^, ^245^, ^246^ A series heterogeneous nanostructure with 2D nanocrystals have been developed by Zhang's group through epitaxial growth method. In 2016, they synthesized heterogeneous nanostructure about controlled growth nanorods arrays on selective facets of 2D nanoplates.^133^ The CuS based nanocrystals were used as the seeds and controlled growth CdS and CdSe nanorods on the different facets of the seeds. Additionally, they also prepared MoS_2_ and MoSe_2_ ultrathin nanosheets on the Cu_2−_ *_X_*S nanowires in an epitaxial manner via cation exchange method.^134^ The performance of obtained heterostructures can be tuned by the loading amount and lateral size of the MoS_2_ and MoSe_2_ ultrathin nanosheets, which can be achieved by the injection of chalcogen precursors. The optimal heterostructure catalysts show a better performance for photocatalytic hydrogen evolution reaction than the single species, indicating the controlled synthesis used various strategies for metal chalcogenides‐based heterostructures is a promising way for new energy applications. Furthermore, they also made several nice works about ultrathin 2D covalent organic framework nanocrystals.^244^ Xie and co‐workers prepared many ultrathin 2D nanosheets through liquid ultrasonic exfoliation of bulk nanomaterials.^79^, ^84^, ^96^, ^97^, ^128^, ^131^ The thickness of the as‐synthesized nanosheets are several atomic layers thick and possess abundant vacancies for both metal and nonmetal. Additionally, their also used excess methods to further control the structure of the obtained ultrathin 2D nanosheets, such as components regulation, electronic structure optimization, and phase shift. The optimal metallic Mn‐CoSe_2_ nanosheets^64^ with subtle atomic arrangement distortion provides excess active edge sites for HER verified by high‐resolution transmission electron microscopy (HRTEM). Further, the DFT results reveal that the lower kinetic energy barrier of adsorbed H atoms after lattice disorder on Mn‐CoSe_2_ ultrathin catalysts benefiting H_2_ evolution. This work gives a new sight for synthesis of the metal chalcogenides‐based nanocrystals by components regulation. Additionally, they fabricated Bi_2_Se_3_ single layers with five‐atom‐thick via a liquid exfoliation strategy.^245^ Those ultrathin nanosheets show increased densities of states (DOS) and carrier mobility, effective phonon scattering, and decreased thermal conductivity for the surface distortion confirmed by EXAFS and DFT results. Based on the optimized properties of Bi_2_Se_3_ single layers, their display a superior ability for thermoelectric energy conversion. Similarly, they carefully synthesized ZnIn_2_S_4_ atomic nanosheets with controllable defect concentrations and studied the influence of electron−hole separation mechanism based on different defcets.^97^ DFT results demonstrate the enhanced charge density and carrier transport with presence of zinc vacancies (V_Zn_), while the experiment data display the ZnIn_2_S_4_ NSs with abundant V_Zn_ exhibit a superior performance for carbon monoxide formation. Xi's group has developed kinds of 2D nanosheets catalysts, such as NiCo_2_O_4_/NiCoLMO heterogeneous NSs,^246^ CuFeS_2_,^107^ CoFe_2_S_4_,^111^ CuCo_2_O_4_,^108^ NiFe_2_O_4_/FeNi_2_S_4_,^247^ Co‐Fe‐N,^248^ Ni‐C‐N,^249^ and M‐NiS_2_ NSs^250^ etc. Recently, they reported metal (Co, Fe, Cu) doped NiS_2_ NSs with optimize electronic configuration and atomic arrangement. After Co doping, the high‐angle annular dark field aberration‐corrected scanning transmission electron microscopy (HAADF‐STEM) image (**Figure** **10**) clearly shows the atomic arrangement change from NiS_2_ to (NiCo)_3_S_2_, but for the homogenous doping the XRD pattern not changed, indicating the Co‐NiS_2_ NSs still is cubic NiS_2_ structure. The electronic structure also changed after doping, the EXAFS, XANES, and XPS all display the enhanced content of e_g_^1^ and Ni^3+^ active sites. The Co‐NiS_2_ NSs exhibit boosting HER performance in alkaline media with low overpotential (η_j = 10_ = 80 mV) and long‐term stability (90 h), while the TOFs for of those NSs are 0.55 and 4.1 s^−1^ at an η of 100 and 200 mV. The DFT calculations suggest with Co‐doped surface Ni‐3d bands are in long‐range order, as electron‐depletion center, while the surface S‐sites decreased the surface adsorption energy for H_2_O and further guarantee boosting HER performance. This work further demonstrates that metal‐doping strategy is an efficient way for development of highly active catalysts.

#### 3.1.3 3D Nanostructure

**Figure 11:**
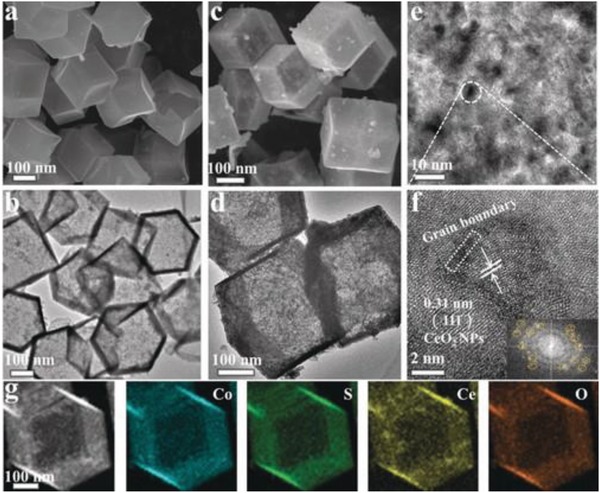
a–d) SEM and TEM images for CoS and 14.6 % CeOx/CoS. e,f) The HRTEM images and corresponding SAED pattern (the inset in (f)) of 14.6 % CeO*_x_*/CoS. g) Elemental mapping images of 14.6 % CeO*_x_*/CoS. Reproduced with permission.^251^ Copyright 2018, Wiley‐VCH.

3D materials with mesoporous and hollow structures in favor of expression for active site, adsorption for intermedia, release and storage of gas act as a promising materials in various field.^221^, ^222^, ^223^, ^224^, ^225^ When applying 3D nanostructures in the electrocatalysis, the efficiency of the reaction will be obviously boosted by the enhanced contact surface, reduced electrons transfer resistance, increased active sites, and easily connected electrolyte and catalysts. Meanwhile, the mesoporous and hollow structures also can promote release of evolved gas and further improve catalytic performance.^120^, ^210^, ^251^, ^252^, ^253^ Tang and co‐workers reported a newly 3D hollow structure based on CeO*_x_* nanoparticles and hollow CoS by surface generation (**Figure** **11**).^251^ The hollow cavity and ultrathin‐wall in the 3D nanostructure provided high surface area and abundant active sites. The optimized CoS/CeO*_x_* 3D hollow nanocatalysts displayed an excellent OER performance with a better overpotential and stability. This work gives a new sight for developing the efficient transition metal chalcogenides (TMSs) through introducing the rare earth ions to adjust the electronic structure of the TMSs. Miao and co‐workers reported a sol–gel method prepared mesoporous FeS_2_ materials with a higher surface area of 128 m^2^ g^−1^.^252^ For the increased number of active sites, the mesoporous FeS_2_ shows an excellent HER electrocatalyst in alkaline media with low overpotential (η_j = 10_ = 96 mV) and long‐term stability. The mesoporous FeS_2_ with boosting HER performance, if they are also active for OER, it may facilitate the development of overall water splitting. Similarly, Geng's groups reported a mesoporous MnCo_2_O_4_ with abundant Mn^IV^ and Co^II^ on the surface, which displayed an excellent performance for reversible oxygen catalysis,^250^ and indicated potential application in renewable energy technologies and devices. Additionally, the unique synthesis method of spray‐pyrolysis can be used to prepare other mesoporous materials. The new synthetic method also should be designed and used to prepare the 3D structure with superior electrocatalytic performance. Carbon coating is another interesting strategy for synthesis the 3D hybrid electrocatalysts. With the enhanced conductivity and protective effect of thin carbon shell, those catalysts show increased activity and stability for kinds of catalytic reactions. Kims's group demonstrates carbon shell coating strategy display different effect with different shell layers. Recently, their prepared N‐graphitic (GN) shell coated nitride‐Ru nanoparticles (NPs) and studied the catalytic performance of Ru NPs with different carbon layers coating.^43^ They found that catalytic activity will significantly reduce when more than three carbon layers coated on the NPs surface for the outermost carbon layer prevent the electron transition to encapsulated metal–core, while the activity and stability of the catalysts will have a high enhance once they are encapsulated in one‐layer carbon shell. This find is very important for synthesis the carbon coating catalysts with controllable layers.

### 3.2 Structure

#### 3.2.1 Phase Change

**Figure 12:**
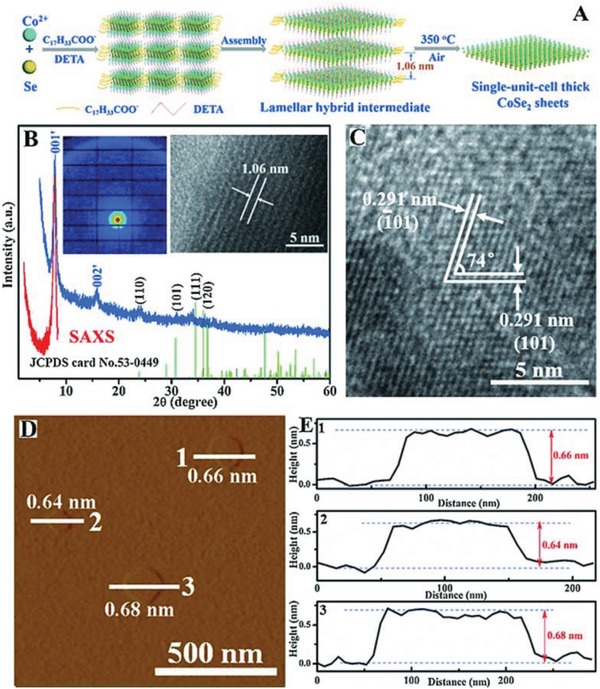
A) Schematic illustration for fabricating thick orthorhombic phase CoSe_2_ sheets. B) SAXS profile (red line and inset left image), XRD pattern (blue line) and the corresponding lateral TEM image for the lamellar hybrid CoSe_2_‐DETA intermediate. C) HRTEM image of the thick orthorhombic phase CoSe_2_ sheets. D) AFM image and E) the corresponding height profiles for the thick orthorhombic phase CoSe_2_ sheets. Reproduced with permission.^254^ Copyright 2015, Wiley‐VCH.

The properties of nanomaterials, such as conductivity, surface adsorption energy, energy band, and so on, will be rapid changed with various crystal phase, which give unexpected enhancement of electrocatalytic activity. Metal chalcogenides with various atomic and electronic structures offer opportunities for fabrication different crystal phase. The MoS_2_ or WS_2_ based materials usually show two different phase: semiconducting trigonal prismatic (2H phase) and metallic octahedral (1T phase), while phase‐transformation strategy is easily created from 2H to 1T with increasing activity for enhanced the electron density in the d orbitals.^31^, ^57^, ^121^ Xie and co‐workers reported the *orthorhombic*‐CoSe_2_ and *cubic*‐CoSe_2_ (**Figure** **12**).^254^ With different phase structure, the different Co—Se bond lengths show different free adsorption energy for both H atoms (H_ads_) and water molecular, revealing the *cubic*‐CoSe_2_ shows dramatically improved activity compared with *orthorhombic*‐CoSe_2_. The experiment and DFT data show that *cubic*‐CoSe_2_ has higher electrical conductivity, optimized adsorption energy, and high efficiency for H_2_ production, suggesting that phase‐transformation engineering is a highly efficient way for designation active electrocatalysts. Chhowalla's group used lithium intercalation method to synthesize WS_2_ NSs with enhanced HER activity.^255^ They found that those 1T phase NSs prepared by exfoliation method have abundant disorder (1T' phase) with hackly W—W bond structure, while exhibit more active performance than pure 1T phase for HER.^255^ As expected, the HER activity of those electrode is quickly reduced if change 1T–2H phase. DFT results demonstrate the strain have a positive effect for Δ*G*_H*_ on 1T‐WS_2_, while the strain can be changed with the increased DOS below the Fermi level (F_E_). Similarly, other works also give the same conclusion under phase conversion from 2H to 1T, the HER activity of exfoliated MoS_2_ will improve.^48^, ^238^, ^256^, ^257^ Those results also agree with Nørskov group data about the energy of H_ads_ on metallic TMDs surface.^258^ Therefore, the phase change should be acting as an effective way for preparing the superior electrocatalysts.

#### 3.2.2 High Index Crystal Face

**Figure 13:**
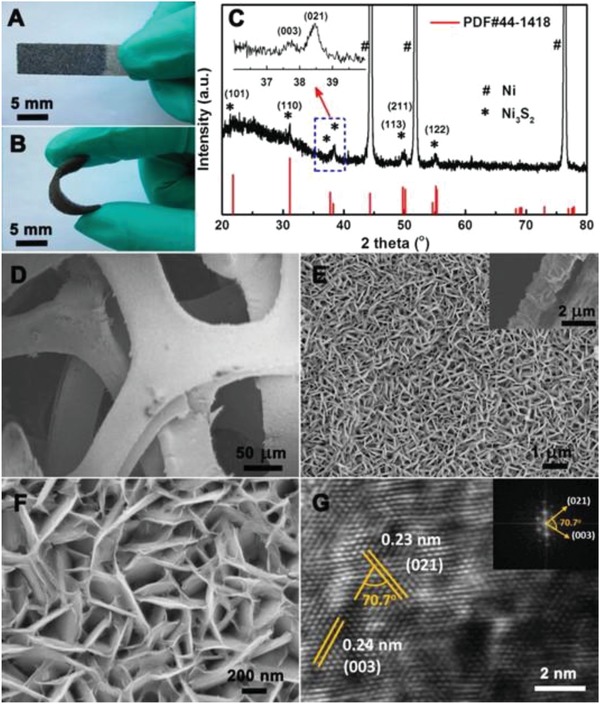
A,B) Photos, C) XRD pattern, and D−F) top‐view SEM images of the Ni_3_S_2_/NF. Inset in (E): side‐view SEM image of Ni_3_S_2_/NF. G) HRTEM image of the Ni_3_S_2_/NF, with the fast Fourier transform image shown in the inset. Reproduced with permission.^67^ Copyright 2015, American Chemical Society.

Generally, nanocrystal with high‐index planes usually exhibits a high density of atomic steps, ledges, and kinks, which can serve as highly active sites for catalysis, energy conversion, and other reactions.^259^ Consequently, develop the nanocrystal with high‐index facets is a potential route for the fabrication of highly effective next‐generation nanocatalysts. The different exposed high‐index facets can be obtained by shape‐controlled syntheses. However, high‐index facets are not stable and often evolve and disappear rapidly during the synthesis or in reaction due to their high surface energy.^107^ Thus, the development of high‐index faceted nanosheets with rich active sites and good structural stability is a block toward our goal of exploring highly efficient electrocatalysts. Yan and co‐workers designed and prepared two kinds of Pd nanoshells with high‐index facets of {730} and {221} through heteroepitaxial growth on high‐index‐faceted Au nanocrystals.^260^ The turnover numbers per surface atom of the high‐index faceted Pd nanoshells have been found to be 3–7 times those of Pd and Au‐Pd core–shell nanocubes that possess only {100} facets in catalyzing the Suzuki coupling reaction. These results open up a potential for the development of inexpensive and highly active metal nanocatalysts. Additionally, there are many researches have an extensive interest in high‐index faceted noble‐metal based nanocrystal. However, due to the high cost and low Earth abundance of those metals, the widely application of those catalysts is limited. Therefore, it is currently a dire need to develop alternative catalysts from Earth‐abundant elements with good activity and durability. Zou's group reported a $\left\{\bar{2}10\right\}$ high‐index faceted Ni_3_S_2_ nanosheet arrays on NF for the first time (**Figure** **13**).^67^ The Ni_3_S_2_/NF with high‐index faceted can serve as a highly active bifunctional electrocatalyst for HER and OER with a Faradaic yield close to 100% and remarkable stability for more than 200 h. More importantly, both the Experimental and DFT results indicate that Ni_3_S_2_/NF's superior catalytic activity can be attributed to the exposed $\left\{\bar{2}10\right\}$ high‐index facets.

#### 3.2.3 Amorphous Structure

**Figure 14:**
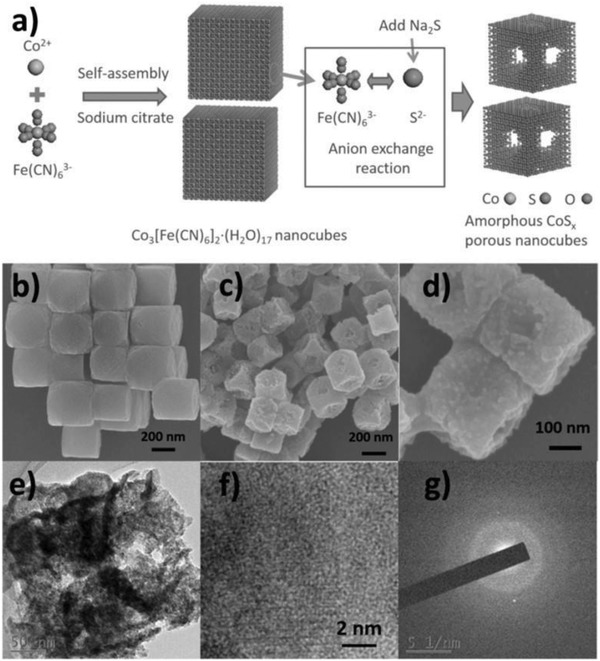
a) Illustration of synthesized process of A‐CoS_4.6_O_0.6_ PNCs. b) SEM image of Co‐Fe PBA precursor. c,d) SEM images at different magnification. e–g) TEM, HRTEM image, and SAED pattern of A‐CoS_4.6_O_0.6_ PNCs. Reproduced with permission.^120^ Copyright 2017, Wiley‐VCH.

Except the crystalline nanomaterials, amorphous structure also has aroused extensive interest in the many field. Unlike the long‐range atomic order of the crystalline solids, the amorphous ones possess only short‐range order over a few atoms. Additionally, the amorphous materials also have unique characteristic, such as abundant defects, isotropic properties (soft magnetic, mechanical, corrosion resistance, electronic, catalytic), metastability.^120^, ^261^, ^262^ However, the researches on amorphous materials still need more study. This is because the internal structure of amorphous materials is so complicated that almost no success has been achieved in building a complete model or systematic theory to represent or describe them. Nevertheless, the less explored amorphous materials are, the more fascinating they are to researchers. Efforts also have been devoted to the research of local atomic environment and ions transport in the amorphous materials. Furthermore, their specific atomic arrangement enables amorphous materials to exhibit high performance in mechanics and catalysis, as well as interesting magnetic properties. Wen and co‐workers reported a novel OER electrocatalyst (A‐CoS_4.6_O_0.6_ PNCs) with superior OER performance in alkaline/neutral medium via doping engineering (**Figure** **14**).^120^ Both experimental and DFT results demonstrate A‐CoS_4.6_O_0.6_ PNCs has the desirable O* adsorption energy and outstanding electrocatalytic activity. Additionally, other work also reported similar phenomenon of the amorphous structure nanomaterials. Zhao and co‐workers reported CuCo_2_S_4_ nanosheets for efficient catalysis ORR and OER.^262^ However, with the formation of surface amorphous oxides, the catalytic performance of CuCo_2_S_4_ nanosheets decreased for the loose of catalytic active sites, which is inevitable in OER.

### 3.3 Electronic Structure Optimization

#### 3.3.1 Defects Engineering

**Figure 15:**
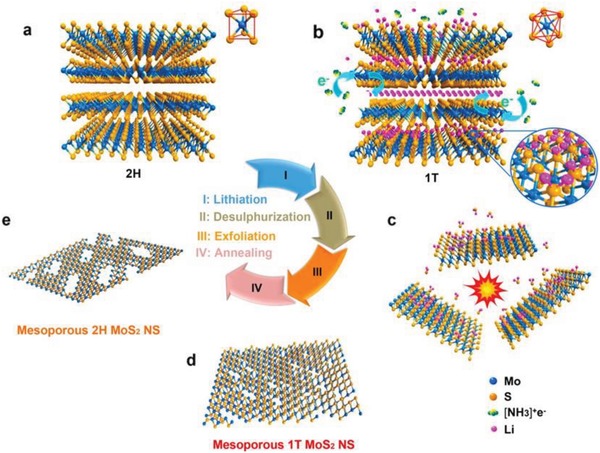
a–e) Schematic illustration of the preparation of mesoporous 1T phase MoS_2_ nanosheets from bulk MoS_2_. Reproduced with permission.^56^ Copyright 2016, American Chemical Society.

The typically involve engineering of those efficient methods for the enhanced catalytic activity of the metal chalcogenides is to increase the percentage of edges exposed, such as MoS_2_ or introduce structural defects to create new active sites (e.g., pores, vacancies, lattice distortion). Jin and co‐workers reported a novel porous (holey) metallic 1T phase MoS_2_ nanosheets by a liquid‐ammonia‐assisted lithiation strategy (**Figure** **15**).^56^ In this research, the authors systematically investigated the contributions of crystal structure (phase), edges, and sulfur vacancies (S‐vacancies) to the catalytic activity toward HER, in which optimal HER catalyst was the porous 1T phase MoS_2_ nanosheets with abundant edges and S‐vacancies. The observation of MoS_2_ edge activity for the HER has opened new avenues for increasing catalyst performance by optimizing the basal phase, edge ratio, and defects. Song group reported vanadium heteroatoms doped pyrite NiS_2_ nanosheets as bifunctional electrodes for both HER and OER.^73^ Notably, the electronic structure reconfiguration of pyrite NiS_2_ is observed from typical semiconductive characteristics to metallic characteristics by engineering vanadium (V) displacement defects, which is confirmed by both experimental temperature‐dependent resistivity and theoretical density functional theory calculations. Furthermore, elaborate X‐ray absorption spectroscopy measurements reveal that electronic structure reconfiguration of NiS_2_ is rooted in electron transfer from doped V to Ni sites, consequently enabling Ni sites to gain more electrons. The metallic V‐doped NiS_2_ nanosheets exhibit extraordinary electrocatalytic performance with overpotentials of about 290 mV for OER and about 110 mV for HER at 10 mA cm^−2^ with long‐term stability in 1 m KOH solution, representing one of the best non‐noble‐metal bifunctional electrocatalysts to date. This work provides insights into electronic structure engineering from well‐designed atomic defect metal sulfide. Recently, more and more researches indicate that the defects play an importance role in electrocatalytic reactions, especially the intrinsic defects. With various new strategies, such as exfoliation, plasma etch, reduction, and so on, were developed to enhance the content of the defects in the obtained nanocrystal. However, the relationship between the content of defects and electrocatalytic activity is still not very clear, and great efforts still need to make an in‐depth study in this field. Afterward, Wang's group reported a new plasma‐engraving strategy for preparing the efficient Co_3_O_4_‐based OER catalysts.^263^ The plasma etched Co_3_O_4_ shows abundant oxygen vacancies and porous nanosheets structure with increased active sites and surface area. Not surprisingly, the plasma‐etched Co_3_O_4_ nanosheets show an OER potential of 300 mV at current density of 10 mA cm^−2^. This plasma technology could also be used to create other superior electrocatalysts thought different plasma gas and precursor, which may open a new sight for fabrication metal compounds with abundant defects. Xie and co‐workers have performed many exciting studies about these defects materials.^74^, ^79^, ^96^, ^97^, ^128^, ^131^ Recently, they prepared ultrathin CoSe_2_ nanosheets with controllable cobalt vacancy (Co*_v_*).^84^ During the ultrasonic exfoliation progress, the diethylenetriamine (DETA) was used to combine Co atoms by coordinate bonds, and then Co*_v_* can be formed by detaching the coordinated Co atoms from the lattice of CoSe_2_. This work also gives a careful characterization about those Co*_v_* by PAS and X‐ray absorption fine structure spectroscopy (XAFS). The DFT results suggest that the Co*_v_* in the ultrathin structure were more favorable for adsorbing H_2_O molecules. The optimized electronic structure and favorable H_2_O adsorption energy made the ultrathin CoSe_2_ nanosheets with abundant Co*_v_* sites performed much better OER activity. Similarly, Zhang's group also reported novel OER catalysts of NiO/TiO_2_ hybrid with abundant nickel vacancies (Ni*_v_*) and oxygen vacancies (O*_v_*).^20^ After abundance Ni*_v_* sites created in the ultrathin NiO/TiO_2_ hybrid nanosheets, the hybrid structure shows changed electronic structure for the created Ni^3+^ sites with ideal orbital occupancy approaching unity (t_2g_^6^e_g_^1^). This metal‐cation‐vacancy strategy may be applied to other OER electrocatalysts, such as phosphides and chalcogenides. Advanced methods should also be further developed (plasma, electron bombardment) and maintained the defects at the atom scale. The defects models of those unique nanostructure are designing and using in further.

#### 3.3.2 Fabrication of Nanointerface

**Figure 16:**
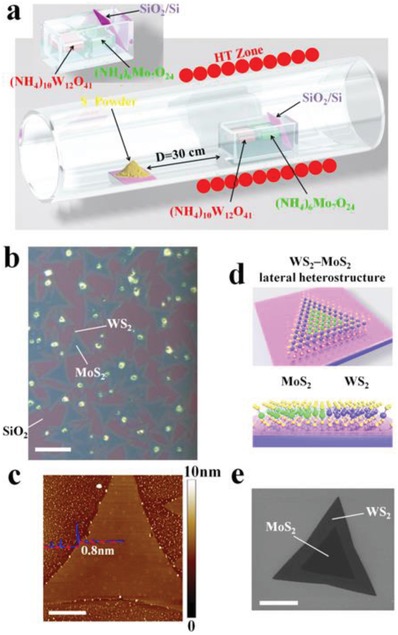
a,d) Schematic illustration of home‐built APCVD setup and WS_2_/MoS_2_ in‐plane heterojunctions. b,c,e) Optical, AFM and SEM image of the WS_2_/MoS_2_ in‐plane heterojunctions. Reproduced with permission.^177^ Copyright 2015, Wiley‐VCH.

Another efficient way for the fabrication of highly active electrocatalysts is developing the heterogeneous nanocrystal with highly active nanointerface, such as metal and metal oxides, metal compound and carbon materials, inorganic substance, and MOF. The electronic structure of those two different components closer to the nanointerface will be changed for the formation of the unique nanointerface. The charge transfer, adsorption energy, and active sites are all optimized after the fabrication nanointerface, resulting in the enhanced electrocatalytic performance. Recently, many works have demonstrated that the important role of the nanointerface in the electrocatalysis.^108^, ^109^, ^110^, ^111^, ^177^ Sun and co‐workers reported the monodisperse Co/CoO NPs on the graphene surface. The obtained heterogeneous nanocrystal shows two clear nanointerface, namely PNs−graphene and Co−CoO.^264^ The strong interaction between the two nanointerface rapidly increases the NPs catalytic performance. Additionally, the optimized core–shell structure with 8 nm Co core and 1 nm CoO shell displays a boosting ORR performance much better than the commercial Pt/C. Transition‐metal chalcogenides, especially the heterogeneous structure, have revealed interesting phenomenon in the quantum spin Hall effect, valley polarization, superconductivity, catalysis, and so on. Xu and co‐workers reported a high‐quality WS_2_/MoS_2_ in‐plane heterostructures by the ambient pressure CVD method (**Figure** **16**).^177^ Advanced technologies were used to studied and verified the WS_2_/MoS_2_ heterostructures, such as TEM, scanning Kelvin probe force microscopy (SKPFM), Raman and photoluminescence spectra. The effects of the built‐in potential of lateral WS_2_/MoS_2_ heterostructures on the significantly different functions of single‐layer WS_2_ and MoS_2_ were quantitatively analyzed using SKPFM measurements. The SKPFM results show that the built‐in electric field and width of each depletion layer are quantitatively estimated for the first time in the WS_2_/MoS_2_ heterostructure. Combining the newly designed synthetic strategy with the quantitative characterization of SKPFM's built‐in potential can be further extended to the heterostructure of other metal chalcogenides. Additionally, Xi's group have been devoted an in‐depth study to the nanointerface electrocatalysts and obtain several bright works.^108^, ^109^, ^110^, ^111^ They have successfully prepared several heterostructures materials, such as oxides and oxides, oxides and chalcogenides, nitrides and carbons, nitrides and oxides. Recently, they reported an interesting work about the in suit fabrication of oxides and chalcogenides heterostructures.^109^ The NiS_2_/CoS_2_−O NWs display boosting reversible oxygen catalysis and the high efficiency can be attributed to the abundant oxygen vacancies and interface porous nanowires structure. Most interestingly, the self‐driven water splitting device made by NiS_2_/CoS_2_−O NWs shows highly efficient and excellent stability. Recently, more and more interesting work about the nanointerface materials have been reported, which also brought several new sights for the nanointerface materials. It should be believed that the research of the nanointerface will have a huge leap in the future.

#### 3.3.3 Stress Regulation

The electrocatalytic activity can be enhanced by optimizing the electronic structure of materials through the stress regulation. Lattice strain, both tensile and compressive stress, can be altered by modifying the distances between different atoms on the surface of the materials. Generally, most stress studies focus on Pt‐based nanomaterials, suggested that 1% lattice strain in those nanomaterials can be introducing the 5d‐band center shifted by ≈0.1 eV,^265^ which can change the surface adsorption energy of Pt‐based nanomaterials. The most common ways for regulating stress are fabricating core–shell structures and metal vacancies in MPt (M: Fe, Co, Ni, Cu, and so on) nanocrystals to form lattice‐mismatch. The shortcoming of those two methods is typically restricted to compressive strain for the larger lattice of Pt compared with other metal cores and instability charges transition between metal atoms in different coordination environments. Therefore, intensive studies have been done to investigate the intrinsic relationship. Thus, new methods that can effectively control both tensile and compressive lattice strain in nanomaterials without introducing additional effects are needed. Recently, a newly strategy was developed to investigate the stress, which is depositing the catalysts onto the flat substrate under physical transformations.^266^ Huang's group reported a desirable result about stress regulation in MPt nanocrystals.^267^ The platinum‐lead/platinum (PtPb/Pt) core/shell nanoplate catalysts with uniform morphology, structure, and thickness were successfully prepared. The PtPb/Pt core/shell nanoplate exhibits large biaxial strains for the unique atoms structure. DFT results reveal that PtPb/Pt core/shell nanoplate displays optimized Pt—O bond strength for the large tensile strains created between edge‐Pt and top (bottom)‐Pt (110) facets. The PtPb/Pt nanoplate shows a superior stability (50 000 cycles) without activity decay, structure, and composition changes, which can be attributed to the intermetallic core and surface layer Pt. Cui and co‐workers controlled the lattice strain of Pt and tuned the catalytic performance for ORR.^268^ In their research, the small Pt NPs (≈5 nm) were carefully deposited on the surface of LiCoO_2_ or Li_0.5_CO_2_ particle (≈500 nm). Then, by controlling the charge and discharge state, applying about 5% compressive strain and tensile strain on the Pt (111) surface, which can be used TEAM to confirm. As a result, the ORR catalytic activity of those Pt NPs has a large change under compression and stretching stain with about 90% or 40% lower. Additionally, the DFT results also show the similar conclusion. This work used Li ions intercalated into or extracted out of battery materials to tune the stress, which provided a new sight in electronic structure regulation by stress for other electrocatalysts. The studies of stress regulation for transition metal chalcogenides also have attracted the attention of researchers. Kuo's research team used 1T‐MX_2_ (M = Mo, W; X = S, Se, Te) as the model studied the strain for HER activity.^269^ In their method, the stress changes of MoS_2_ and WS_2_ were above 2%, and the stress changes of MoSe_2_ and WSe_2_ were above 6%, respectively. The application of strain increases the DOS near F_E_, reduces the free surface adsorption energy of Δ*G*_ads,H_, and further enhances HER activities. The author proposes to HER on 1T‐MX_2_ tension‐strain‐based adjustments, which can serve as a cheap alternative to noble catalysts such as Pt. However, the researches about stress regulation on metal chalcogenides nanocrystal usually only used doping method, such as Co, Ni, or Fe. Usually, the energy of Δ*G*_ads,H_ for MoS_2_ promoted for the changed stress after doping. Those results show that the Δ*G*_ads,H_ on doped metal chalcogenides is dramatically affected by the stress changes. More importantly, the new method should be developed to tune the stress of those metal chalcogenides and obtain the nanocrystals with superior electrocatalytic activity.

## 4 Concluding Remarks and Outlook

In summary, the unique properties of metal chalcogenides nanomaterials, such as ideal atomic arrangement, high electrical transport, easily regulated structure, and large‐scale synthesis, have great research in many fields. As fascinating materials, kinds of synthetic methods and optimized strategies have been designed for synthesis those metal chalcogenides nanocrystals, in order to obtain more active and stable metal sulfide‐based nanomaterials. Additionally, the hybrid engineering based on metal chalcogenides gives chance for developing novel functional composites with other materials, such as metals, oxides, nitrides, chalcogenides, and carbides. However, the controlling preparation of those heterostructures nanocrystals in finely regulates the content of different components still remains a challenge. Significantly, these results present amazing performance in overall water splitting, like MoS_2_, Ni_3_S_2_, CoS_2_, and other metal chalcogenides‐based nanocrystals.

Recently, more and more works indicate that microstructure regulation on atomic scale plays an important role in the formation of more active nanomaterials for various application. Meanwhile, with the development of single‐atom metal catalysts, the newly metal chalcogenides with more active sites, increased exposed atom and atomic efficiency should be designed under new sight to low the overpotential and enhance the stability of water splitting. Herein, we summarized several atomic scale strategies for preparation of more boosting electrocatalysts for overall water splitting, like phase change, atomic nanointerface fabrication, atomic vacancies, and defects. However, it should be noted that the researches of functional components base on metal chalcogenides for overall water splitting is still takes a lot of effort. Currently, new synthetic methods are in urgent need of design to prepare metal chalcogenides with other unique structure. Previous studies suggest that the electrocatalytic performance of various metal chalcogenides synthesized through traditional method still lower than that of Pt, Ir, Ru, and other noble metal‐based catalysts for HER, OER, ORR, water splitting, and other catalytic reactions, which cannot be a promising alternative candidate for industry application. One of the future developments of metal chalcogenides lies in the synthesis of metal chalcogenides based on its structure change after electrocatalytic reactions. The structure of electrocatalysts must be changed for the electrons and protons transform between electrocatalysts and electrolyte during the electrocatalytic reactions. Therefore, it is important to give a clear proof for the structure and active species of metal chalcogenides after the electrocatalytic reactions, and then design efficient synthetic routes to prepare those high‐efficient metal chalcogenides for water splitting.

Although the aforementioned challenges really exist in metal chalcogenides, the development of metal chalcogenides still has a tremendous progress. With the development of technology and synthesis strategy, the characterization and controlled synthesis for metal chalcogenides exhibit more accuracy and higher success rate. The surface‐active site, electroconductivity, reaction properties, new morphology, and structure of metal chalcogenides have been successfully realized. Meanwhile, the relationship of defect, stress, interface, and electronic structure with the activity of various reactions have been extensively studied by many researchers. Furthermore, the metal chalcogenides display a better performance in electrocatalysis, lithium and lithium–sulfur batteries, and so on. Thus, it is necessary to develop precision way for metal chalcogenides to obtain pure phase, active interface, exposed active surface, optimized electronic structure, enhanced electronic conductivity, and fabricate heterostructure with other nanocrystals, in order to further increase their activity and stability for HER, OER, ORR, water splitting, and other applications. Additionally, the metal chalcogenides based various devices should be developed, and the reaction mechanism of those metal chalcogenides on different interfaces with different active sites should be further researched in those devices. The practical application of metal chalcogenides in various fields requires substantially improved environment protecting property and stability, thus calling for further research focus on the security and long‐term stability of metal chalcogenides.

## Conflict of Interest

The authors declare no conflict of interest.
